# ROS-lowering doses of vitamins C and A accelerate malignant melanoma metastasis

**DOI:** 10.1016/j.redox.2023.102619

**Published:** 2023-02-02

**Authors:** Muhammad Kashif, Haidong Yao, Sarah Schmidt, Xue Chen, Michelle Truong, Elin Tüksammel, Yiran Liu, Martin O. Bergo

**Affiliations:** a Department of Biosciences and Nutrition, Karolinska Institutet, SE-141 83, Huddinge, Sweden; b Center for Hematology and Regenerative Medicine, Department of Medicine Huddinge, Karolinska University Hospital, SE-141 86, Huddinge, Sweden

## Abstract

Oxidative stress is a barrier of migration and metastasis for malignant melanoma cells. Consequently, reducing oxidative stress with the antioxidant *N*-acetylcysteine (NAC) stimulates melanoma cell migration in vitro and metastasis in vivo. However, it is not yet known whether the NAC effect is shared with other antioxidants. Here, we screened 104 redox-active compounds and identify 27 that increase migration of human malignant melanoma cells in two doses. Validation experiments in four cell lines and four drug doses resulted in a list of 18 compounds which were ranked based on their ability to increase migration and reduce ROS levels; vitamin C (VitC) ranked as number one, followed by the vitamin E analogue Trolox and several carotenoids and Vitamin A–related compounds. Four diet-relevant compounds from this list—VitC, β-carotene, retinyl palmitate, and canthaxanthin—were selected and found to accelerate metastasis in mice with BRAF^V600E^-driven malignant melanoma. Genomics analyses revealed that the transcription factor BACH1 is activated following antioxidant administration and knockout of *Bach1* in mouse melanoma cells reduced lymph node and liver metastasis in xenograft mouse models. We conclude that a broad range of antioxidants accelerate melanoma migration and metastasis and that BACH1 is functionally linked to melanoma metastasis in vivo.

## Highlights

• Common diet-relevant antioxidants accelerate melanoma cell migration and metastasis.
• BACH1 is functionally linked to melanoma metastasis in vivo.

## 1 Introduction

Malignant melanoma accounts for a low percentage of all cancer diagnoses but the incidence has increased dramatically over the past half-century, and while immunotherapy and checkpoint inhibitors have reduced the overall lethality of this cancer, the 5-year survival rate for stage IV melanoma remains below 30% [1]. Thus, the need for increasing the molecular understanding of factors that influence melanoma risk and progression remains high.

Recent studies have revealed that oxidative stress limits the ability of melanoma cells to metastasize [2]. This finding can explain why incubating human malignant melanoma cells with the antioxidants *N*-acetylcysteine (NAC) and the Vitamin E (VitE) analogue Trolox increase cell migration and cell invasion; and why supplementing the drinking water with NAC accelerates metastasis in mouse models of malignant melanoma ([2,3]).

Similar studies revealed that administering ROS-lowering doses of NAC in the drinking water or VitE in the food accelerates metastasis rates in mice with KRAS^G12D^-induced lung cancer; this effect is mediated by the redox-sensitive transcription factor BACH1 (BTB domain and CNC homolog 1) ([4,5]). However, it is not yet clear whether BACH1 is activated in malignant melanoma cells and contribute to metastasis. To overcome or avoid oxidative stress associated with metastasis, tumor cells can activate endogenous NF2L2 (NRF2)-controlled antioxidant pathways and by metastasizing through lymph—where oxidative stress levels are low—rather than blood ([5,6]).

In contrast to data showing that ROS and oxidative stress limits tumor progression, many studies reveal that certain forms of ROS and oxidative stress–inducing conditions can stimulate cancer cell migration and tumor progression ([7,8]). Yet, it is not yet clear whether antioxidants and redox-active compounds other than NAC and Trolox [3]—including diet-relevant carotenoids, vitamins, and retinols—would accelerate melanoma cell migration and metastasis. To address this issue, we screened a library of 104 redox-active compounds for their ability to increase migration of human malignant melanoma cells; we first identified a ranked list of 18 compounds and then selected four that were evaluated in a melanoma mouse model. We also performed genomics analyses to define molecular alterations associated with antioxidant administration in melanoma cells.

## 2 Materials and methods

### 2.1 Cell culture

Human malignant melanoma cell lines SK-MEL-30 and IPC-298 were obtained from the German Collection of Microorganisms and Cell Culture; cell lines SK-MEL-3 and A-375 were from American Type Culture Collection (ATCC). The mouse malignant melanoma cell line B16–F1 was from ATCC. Cell lines tested negative for mycoplasma and were cultured in DMEM (Dulbecco's modified Eagle's medium) GlutaMax High Glucose (4.5 g/L, 10,569–010) supplemented with 10% fetal bovine serum (26,140,079, ThermoFisher) and 1% penicillin/streptomycin (15,070,063, ThermoFisher).

### 2.2 Bioactive redox compounds

We used the Screen-well Redox library (BML-2835, Enzo Biochem Inc.) containing 84 compounds and the following 20 bioactive redox compounds from Sigma: Afungin (S545791), Balsalazide disodium (B5438), Catechin gallate (C0692), Chlorogenic acid (C3878), Delphinidin chloride (43,725), Diosmin (D3525), Epicatechin (E1753), Ellagic acid (E2250), Epicatechin from green tea (E4018), Epigallocatechin (E4268), Fucoxanthin carotenoid (F6932), Gallic acid (G7384), Gallocatechin (G6657), Ginkgolide B (G6910), Hesperidin (H5254), Kaempferol (K0133), Luteolin (L9283), Neochlorogenic acid (94,419), *p*-Coumaric acid (C9008), and Rutin hydrate (R5143).

### 2.3 Cell migration

Cell migration was measured using a wound healing assay in the IncuCyte ZOOM system (Essen BioScience). The cells were seeded in 96-well image-lock plates (4379, Essen BioScience) for 24 h. After 24 h a scratch was made in each well using WoundMaker (Essen Biosciences) which simultaneously creates identical scratches in all wells. Dead cells and debris were removed by washing each well with media 3 times; 200 μl of fresh medium containing the different compounds was then added to each well.

### 2.4 *Trans*-well migration and invasion assays

*Trans*-well migration assays were performed using inserts with a 6.5-mm, 8.0-μm-pore membrane. Cells were suspended in medium containing 1% FBS and added to the upper chamber; the bottom chamber contained medium containing 30% FBS. For invasion assays, cells were seeded in chambers precoated with Matrigel (1:30 dilution) and incubated for 6–7 h. Remaining cells in the upper chamber were then removed with a humidified cotton swab and migrating/invaded cells on the other side of the membrane were fixed with paraformaldehyde, stained with crystal violet, and photographed under a brightfield microscope (Carl Zeiss AB). ImageJ was used to quantify the area covered by cells.

### 2.5 ROS measurements

Cellular ROS levels were measured with the H2DCFDA (H2-DCF, DCF) kit (D399, ThermoFisher). Cells were incubated with compounds, washed with PBS, resuspended in PBS containing 10 μM DCF, and incubated for 1 h at 37 °C. Fluorescence was recorded with a Synergy multimode reader (BioTek).

### 2.6 Screen and ranking procedure

From results of the first screen of 104 redox-active compounds, candidates for further studies were selected based on their ability to increase migration of SK-MEL-30 cells in both doses tested. In the second screen compounds were tested in 4 doses in 4 cell lines, and from the results compounds were selected based on scoring 0 or 1 for three parameters: whether a compound i) increased migration (yes = 1, no = 0; max 16 points); ii) decreased ROS levels (max 16 points); and iii) produced dose-dependent effects on migration and ROS (max 16 + 16 = 32 points); the scores for each parameter were added, and the final sum was used for ranking the 18 most potent migration-enhancing compounds whose effect was associated with reduced ROS levels and thus were functioning as antioxidant compounds. Four compounds were then rationally selected from the 18 based on their relevance to human nutrition and were tested for their ability to influence cell invasion in vitro and tumor progression and metastasis in vivo.

### 2.7 Malignant melanoma mouse model and compound dosing and administration

Two-day-old *Braf*^CA/+^*Pten*^fl/fl^Tyr-*Cre*^+/0^ mice on a mixed genetic background (129SV and C57BL/6) were painted on the flank skin with 4-hydroxytamoxifen (T5648, Sigma) to trigger Cre expression in melanocytes and activate BRAF^V600E^ expression and inactivate PTEN expression [9]. Three weeks later, when small nevi were visible on the skin, littermate mice were randomized to cages where they received either control R34 chow or R34 supplemented with β-carotene, retinyl palmitate, or canthaxanthin (Lantmännen); VitC was added in the drinking water. Antioxidant compounds were delivered in two doses: for β-carotene and retinyl palmitate, the daily recommended allowance (RDA) for mice contained in normal R34 chow is 7.2 and 6.6 mg/kg, respectively; this dose was increased 5 and 50 times to 36 and 360 mg/kg for β-carotene and 33 and 330 mg/kg for retinyl palmitate; Canthaxantine, which is a non-essential keto-carotenoid used as a food-coloring agent in salmon and egg yolk, was added at the same doses as β-carotene. For VitC, which is not present in the R34 chow (because mice, unlike humans, can synthesize VitC), we based the doses on the RDA for humans, 90 mg/day for a 70 kg adult man (i.e., 1.286 mg/kg/day). By multiplying this daily dose with 12.3 to compensate for the faster mouse metabolism [10], we ended up with a tentative daily dose for mice of 15.82 mg/kg/day; and like the carotenoids, we increased this dose 5 and 50 times to 80 and 800 mg/kg/day. The final dose in the drinking water was based on an observed daily water consumption per mouse of 10 ml and a mean body weight of 25 g for males and 18 g for females. The experiment end point was when mice became listless because of primary tumor burden or when primary tumors ulcerated. Mouse experiments were approved by the Research Animal Ethics Committees in Gothenburg and Linköping, Sweden.

### 2.8 RNA sequencing

Total RNA was extracted using RNeasy Plus Mini kit (74,136, QIAGEN) and paired end sequencing was done with an Illumina HiSeq instrument. Sequencing reads were analyzed for quality by FASTQC and were then mapped to the human genome assembly GRCh38.83 with STAR Aligner (version 2.5.0a) [11], and genes were quantified with the featureCounts program in the Subread package (1.5.2) [12]. DESeq2 was used to tests for differential expression of genes identified in the RNAseq analyses [13]. The differentially expressed genes were considered significant if P ≤ 0.05. g:Profiller was used for enrichment analyses and significant enrichments were sorted based on p values [14].

### 2.9 Generation of *Bach1* knockout mouse melanoma cells

CrRNA oligonucleotide 5′-GCTATGCACAGAGGACTCGT-3′ complementary to sequence in exon2 of mouse *Bach1* and its complementary oligonucleotide—both containing 3′-overhangs complementary with the linearized GeneArt-CRISPR Nuclease Vector (Life technologies, A21174)—were annealed and cloned into sequences downstream of the human (h) U6 promotor. In the resulting vector hU6 drives sgRNA transcription and CMV drives bicistronic expression of Cas9 and orange-fluorescent protein (OFP) linked by P2A. The single vector (2 μg) was transfected into B16–F1 mouse melanoma cells (200,000 cells/well in 6-well plates) using jetPRIME (Polyplus) for 4 h. After 48 h transfected cells were enriched by fluorescence activate cell sorting (FACS); 384 sorted cells were seeded as single cells into 96-well plates. The cells were allowed to grow; the medium was changed on day 12; and live clones were split and expanded for cryopreservation and DNA extraction 14–28 days after single cell seeding. Genomic DNA from the clones was extracted with the QIAGEN DNeasy blood & tissue kit (69,506). The *Bach1* knockout event was detected by PCR using three primers: two primers flanking exon 2 (forward: 5′-CCTGTGTCAGAGACAAGTACA-3’; reverse: 5′-CCTCCTCGGGAGACTAGGGCT-3′) and a third indel-detecting primer (forward: 5′-gtttcttGAGTGCGGTATTTGCCTACGA-3’ (gttctt, pigtail sequence). For *Bach1* wildtype or heterozygous clones, this PCR amplifies 490-bp and 310-bp wildtype fragments; for *Bach1* homozygous knockouts the 310-bp fragment will not be amplified due to the indels. One pair of *Bach1*^+/+^ and *Bach1*^−/−^ clones was confirmed by Sanger sequencing and western blots and used in experiments. Sequencing was performed on PCR-amplified and cloned fragments spanning the indel. Western blots were performed as described [4] using primary antibodies recognizing BACH1 (1:1000, sc-271,211, Santa Cruz Biotechnology) and ACTIN (1:1000, A2228, Sigma-Aldrich).

### 2.10 Statistics

Data are mean ± SEM. For statistical analyses, we used the R program; log-rank test was used for survival analyses; Wilcoxon Mann-Whitney test was used when comparing only two groups; and one-way ANOVA was used for all other comparisons. Experiments were repeated 2–4 times unless stated otherwise.

## 3 Results

### 3.1 First screen of 104 redox-active compounds identifies 27 that increase migration of human malignant melanoma cells

**Fig. 1:**
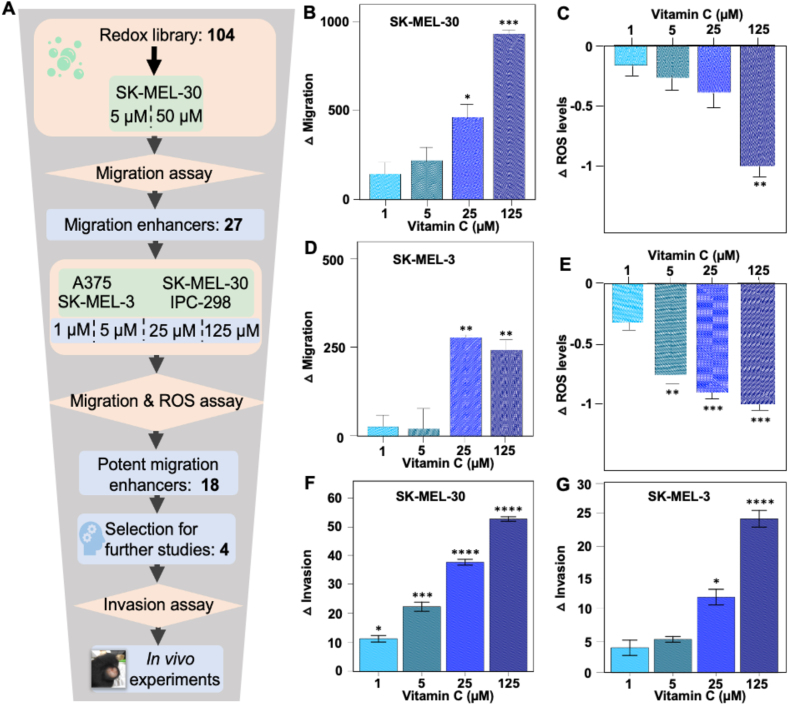
**Vitamin C in ROS-lowering doses increases migration and invasion of *NRAS-* and *BRAF-*mutant human malignant melanoma cell lines.** (**A**) Schematic of screening strategy. Redox compounds (n = 104) were incubated in two doses in malignant melanoma cell line SK-MEL-30, whose migration capacity was quantified using the IncuCyte ZOOM System. Based on migration profiles (shown in Supplementary Figs. 1 and 2) a subset of 27 migration-enhancing compounds were selected—based on their ability to increase migration in both doses—and next evaluated in four doses in four malignant melanoma cells lines for their ability to increase cell migration and reduce ROS levels. The 18 most potent migration-enhancers were ranked based on four parameters (shown in Table 1). Finally, 4 high-ranked diet-relevant compounds (Vitamin C, β-carotene, canthaxanthin, and retinyl palmitate) were selected and evaluated for their ability to influence cell invasion in vitro and malignant melanoma metastasis in vivo. See Material and Methods for further details. (**B**, **C**) Migration (B) and ROS levels (C) of *NRAS*-mutant cell line SK-MEL-30 following incubation with vitamin C for 24 h. (**D**, **E**) Migration (D) and ROS levels (E) of *BRAF*-mutant SK-MEL-3 cells incubated with vitamin C for 24 h. (**F**, **G**) Invasion of SK-MEL-30 (F) and SK-MEL-3 (G) cells incubated with vitamin C for 24 h. Data in panels B–G are expressed as the change (delta) in migration/invasion and ROS levels induced by VitC compared with DMSO control. Data are mean and standard error of quadruplicate analyses. *P < 0.05, **P < 0.01, ***P < 0.001, ****P < 0.0001.

We first performed a screen with a library of redox-active compounds and then two series of validation experiments with selected compounds (Fig. 1A). We screened 104 compounds at two concentrations, 5 and 50 μM, in human malignant melanoma cells (SK-MEL-30) and quantified their ability to close a scratch wound generated mechanically in 96-well plates using the Wound Maker/IncuCyte ZOOM system. As expected, the compounds produced a wide range of effects on cell migration from potent migration enhancing to no effect to potent migration suppression (Supplementary Figs. 1 and 2). Twenty-seven compounds were selected based on their ability to close the wound more efficiently than DMSO controls in both drug concentrations (Supplementary Figs. 1 and 2). Migration suppressors (i.e., those that reduced migration in both concentrations) included many compounds that displayed toxicity and were not studied further here.

### 3.2 Second screen identifies 18 reproducibly migration-enhancing redox-active compounds whose effects are associated with reduced ROS levels

Next, the 27 migration-enhancing compounds were validated at four concentrations, 1, 5, 25, and 125 μM, in four human melanoma cell lines, SK-MEL-30 and IPC-298 which harbor *NRAS* mutations, and SK-MEL-3 and A-375 which harbor *BRAF* mutations, using the IncuCyte system. In parallel experiments, ROS levels were measured in the same cell lines and drug concentrations. The 18 highest ranked migration enhancers were identified based on scoring 0 or 1 for three parameters: whether a compound i) increased migration (yes = 1, no = 0; max 16 points (i.e., one score each for 4 cell lines × 4 doses = 16)); ii) decreased ROS levels (max 16 points); and iii) produced dose-dependent effects on migration and ROS (max 16 + 16 = 32 points). We thus identified 18 ranked compounds with number one showing the most potent and reproducible ability to dose-dependently increase migration and reduce ROS levels (Table 1). Ascorbic acid (VitC) was ranked number one and its effects are shown in Fig. 1, B–E.

**Table 1:** Eighteen ranked migration-enhancing and ROS-reducing compounds from the screening of 104 redox-active compounds. Ranking of 18 redox-active compounds with the most reproducible and potent ability to increase migration and reduce ROS levels in human malignant melanoma cell lines from the screening strategy and follow-up experiments described in Fig. 1A.

Screen rank	Common name	Other name	Increased migration (max = 16)	Reduced ROS (max = 16)	Dose-response (max = 32)	Total (max = 64)
1	Vitamin C	Ascorbic acid	14	9	19	42
2	Vitamin E	Trolox	14	10	17	41
3	Protocatechuic acid	3,4-Dihydroxybenzoic acid	13	14	13	40
	Probucol	Lorelco, Biphenabid	14	10	16	40
4	β-Carotene	Provitamin A	9	8	12	29
5	D609	Tricyclodecan-9-yl-xanthogenate	12	9	6	27
6	GSH	Glutathione	10	6	6	26
7	Canthaxanthin	β-Carotin-4,4′-dione	11	7	6	24
8	Seratrodast	(±)-7-(3,5,6-Trimethyl-1,4-benzoquinon-2-yl)-7-phenylheptanoic acid	9	5	3	17
9	Cumene hydroperoxide	*α,α*-Dimethylbenzyl hydroperoxide	13	1	0	14
	Selenomethionine	Seleno-l-methionine, (*S*)-2-Amino-4-(methylseleno)butyric acid	9	5	0	14
	Tert-butylhydroquinone	NSC 4972	7	7	0	14
10	Paeonol	Resacetophenone 4-*O*-methyl ether	4	9	0	13
	Ferulic acid	4-Hydroxy-3-methoxycinnamic acid	6	7	0	13
11	Ibuproxam	N-Hydroxy-2-(4-isobutylphenyl)propanamid	4	8	0	12
	Esculetin	Cichorigenin	7	5	0	12
12	Retinyl palmitate	Vitamin A palmitate	5	5	0	10
	Ibedenone	2-(10-Hydroxydecyl)-5,6-dimethoxy-3-methyl-1,4-benzoquinone	6	4	0	10

### 3.3 Vitamin C, β-carotene, and two additional vitamin A-related compounds accelerate malignant melanoma metastasis in vivo

**Fig. 2:**
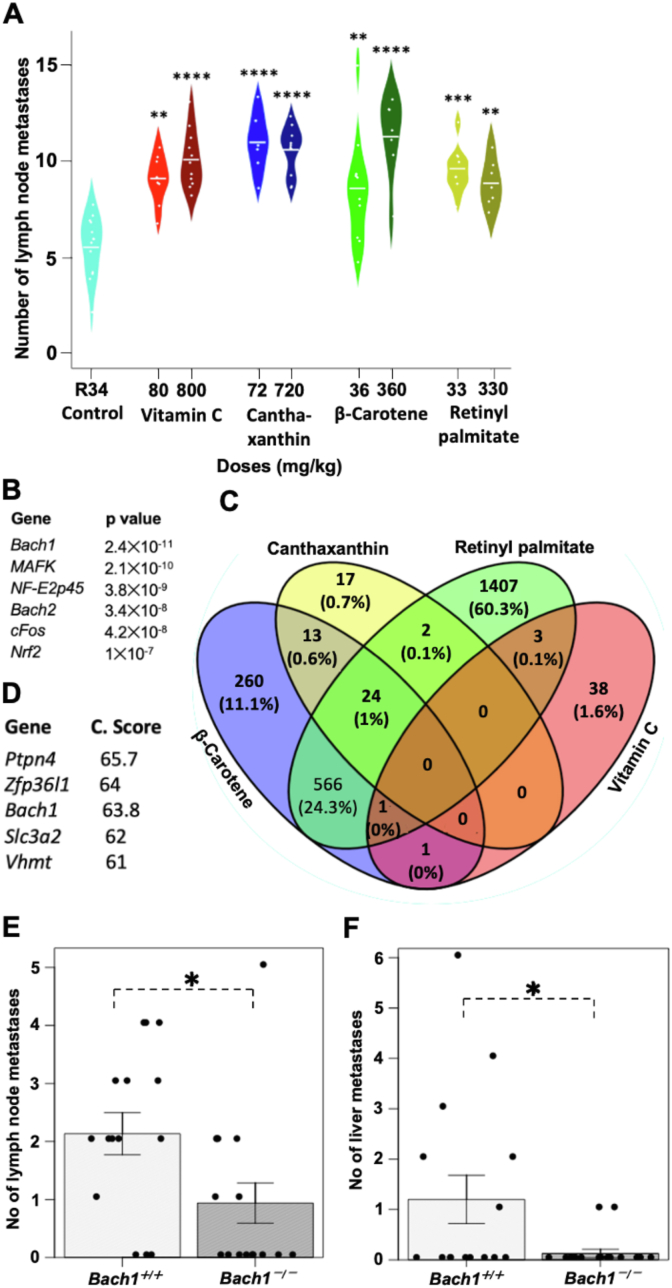
**Common antioxidants increase authentic metastasis in mice with BRAF**^**V600E**^**-induced malignant melanoma; the increased invasiveness is strongly associated with BACH1**. (**A**) Lymph node metastases in *Braf*^CA/+^*Pten*^fl/fl^Tyr-*Cre*^+/0^ mice painted with tamoxifen at 2 days of age and randomized at weaning to receive: control food and drinking water; drinking water supplemented with vitamin C; or food supplemented with canthaxanthin, β-carotene, or retinyl palmitate. Each compound was administered in two different doses as indicated. (**B**) Analyses of RNAseq data from four human malignant melanoma cell lines incubated for 24 h with each of the four antioxidants from panel A. Shown are transcription factors whose motifs were most enriched in promoters of differentially expressed genes (DEGs). (**C**) Venn diagram showing the number and percentage DEGs in response to the four compounds shown in panel A. (**D**) LINCS database analysis showing genes that when overexpressed produced the highest connection (C) scores—i.e., the highest degree of gene expression signature (GES) similarity—compared with the GES observed in nine different human cancer cell lines (including melanoma) following incubation with ten different antioxidants (fraxetin, ascorbic acid, rutin, ascorbyl palmitate, vitexin, mesna, celastrol, seciosolariciresinol, esculin and coumaric acid). (**E, F**) Lymph node (E) and liver (F) metastases detected in mice injected intravenously with *Bach1*^+/+^ and *Bach1*^−/−^ B16F10 mouse malignant melanoma cells. *P < 0.05, **P < 0.01, ***P < 0.001, ****P < 0.0001.

Next, we rationally selected four compounds based on their role in human nutrition. VitC, β-carotene, and retinyl palmitate are essential food nutrients and frequently used in supplements; canthaxanthin is a keto-keratinoid used as a food-coloring agent in salmon and egg yolk. We confirmed that their ability to enhance cell migration extended to cell invasion, i.e., migration through Matrigel in *trans*-well assays (the data for VitC is shown in Fig. 1F and G). To define the impact of the four compounds on authentic malignant melanoma metastasis in vivo, we used *Braf*^CA/+^*Pten*^fl/fl^Tyr-*Cre*^+/0^ (BPT) mice. Painting the skin of BPT mice two days after birth activates expression of BRAF^V600E^ and inactivates PTEN expression in melanocytes and leads to primary malignant melanomas that metastasize to lymph nodes ([3,15]). At weaning, when small nevi are visible on the skin, the mice were randomized to control food and drinking water; water supplemented with VitC; or chow supplemented with Canthaxanthin, β-carotene, or retinyl palmitate—each given in two doses (the motivation and calculation of doses are described in detail in Materials and Methods). As expected from previous studies with NAC [3], the compounds did not influence primary tumor growth, but consistently and significantly increased metastasis (Fig. 2A and Supplementary Fig. 3). The increase in metastases was 1.6–2-fold and was similar with both doses for all four compounds.

### 3.4 BACH1 is associated with the response to antioxidants in melanoma cells and is required for efficient melanoma metastasis

We performed RNASeq of human malignant melanoma cell lines incubated with each of the four compounds and analyzed differentially expressed genes (DEG) of treated cells versus DMSO controls. Overall, the promoters of DEGs were strongly enriched for motifs recognized by BACH1 and related redox-responsive transcription factors (Fig. 2B). As expected, DEGs of β-carotene and retinyl palmitate overlapped significantly (24.3% of all DEGs) whereas carotenoids and Vitamin C shared only 5 DEGs (Fig. 2C). Further analyses revealed a strong enrichment for NF2L2 (NRF2)-related pathways and increased expression of genes involved in GSH production and regeneration in antioxidant-treated cells (Supplementary Fig. 4A and B). We also used the LINCS database to compare the gene expression profiles triggered by overexpression of a given gene (in a genome-wide fashion) in nine human cancer cell lines, with the gene expression profiles obtained when these 9 cell lines were incubated with either of ten compounds characterized as antioxidants. The analysis produces connection scores based on the similarity between the profile observed with the overexpressed gene and the profile observed when the same cells are incubated with the compounds. The gene expression profile observed when BACH1 is overexpressed showed the third highest connection score with the profiles observed following incubation with the ten antioxidants (Fig. 2D). Thus, two unbiased genomics analyses identify BACH1 as a gene and protein associated with antioxidant administration in human malignant melanoma and other cancer cells.

To define the functional importance of BACH1 in melanoma metastasis, we used CRISPR/Cas9 approaches to inactivate *Bach1* in mouse B16–F1 melanoma cells (Supplementary Fig. 4, C–E). We isolated clonal lines of *Bach1*^+/+^ and *Bach1*^−/−^ and injected them in the tail vein of syngeneic mice. As expected from previous studies in lung cancer cells, *Bach1* deficiency did not influence mouse survival, but substantially reduced metastasis to lymph nodes and liver (Fig. 2E and F and Supplementary Fig. 4F).

## 4 Discussion

In this study, we find that a wide range of redox-active compounds can increase migration of human malignant melanoma cells, and that four selected diet-relevant compounds—VitC, β-carotene, retinyl palmitate, and canthaxanthin—accelerate malignant melanoma metastasis in vivo.

Although earlier studies established that NAC and Trolox increase melanoma cell migration and metastasis [3], only Trolox was identified in the screen. The earlier studies used 200 μM NAC whereas 5 and 50 μM were used in the current screen. Yet the effect of the top-ranked compound VitC on metastasis was similar in magnitude as the published findings with NAC (up to 2-fold). The simplest interpretation of this result is that there is a threshold dose in vivo which is easily reached by dietary supplementation after which there is no further increase in metastasis. This argument is supported by the finding that the 10-fold disparate doses for each compound produced similar increases in metastasis.

The ability of the 18 compounds from the second screening to increase melanoma cell migration was associated with reduced ROS levels indicating that the compounds can be classified as antioxidants. Because the majority of the 104 redox-active compounds have been characterized as having antioxidant properties in the literature, our data suggest that most antioxidant compounds cannot increase melanoma cell migration. Indeed, only about one third of the compounds had this capacity in the first screen; most compounds reduced cell migration. We speculate that a substantial proportion of those migration suppressors caused cell toxicity which led to an apparent reduction in migration; but this speculation will have to await confirmation in future studies. In those studies, it will be interesting to determine whether non-toxic migration suppressors act as prooxidants or antioxidants.

Previous studies established that NAC and VitE can accelerate melanoma and lung cancer metastasis ([3,4,16]). Our current finding that also VitC and the two Vitamin A-related compounds β-carotene and retinyl palmitate can accelerate melanoma metastasis suggest that several antioxidant compounds relevant to the human diet have the capacity to spread tumors. The data may also be important from a societal perspective as all these compounds are frequently used in dietary supplements and in skin care products.

It is important to note that although these compounds can increase human malignant melanoma cell migration and invasive properties in vitro, there is no evidence that neither dietary nor topical antioxidants influence malignant melanoma metastasis in vivo in humans. But regardless, we propose that these data, along with previous experimental and clinical studies discussed above, should inform the discussion on nutritional guidelines for cancer patients and people with increased cancer risks: the risks associated with the use of these compounds as supplements clearly outweigh any potential benefits for these groups.

Decades ago, three large, randomized, placebo-controlled studies on antioxidant supplementation and cancer risks had to be stopped early because participants taking β-carotene or VitE were diagnosed with lung or prostate cancer to a greater extent than those receiving placebo ([[17], [18], [19]]). Although there are several potential explanations behind the increased cancer diagnoses, one would be that the trials included from the start varying proportions of participants with undiagnosed tumors whose progression increased in participants receiving antioxidants, thus prompting an earlier diagnosis.

Two complementary genomics analyses revealed a pivotal role for the transcription factor BACH1 in the response to antioxidant administration. On one hand, the finding that a redox-sensitive transcription factor is involved in an antioxidant response is not particularly surprising. But on the other hand, knockout of *Bach1* significantly reduced melanoma cell metastasis to lymph nodes and liver in in vivo experiments—which means that BACH1 can be functionally tied to metastasis in several cancer forms ([4,5]). Future studies should focus on defining the impact of *Bach1* inactivation on endogenous malignant melanoma development and metastasis, for example in *BPT* mice, and compare underlying mechanisms and signaling pathways with those identified in lung cancer.

Finally, these studies reinforce the idea that cancer cells can be killed by pro-oxidant rather than antioxidant strategies, and future studies should be able to identify cancer vulnerabilities that would allow for the development of drugs that can increase oxidative stress to toxic levels specifically in cancer cells [20].

## Declaration of competing interest

The authors declare no competing interests.

## Data Availability

Data will be made available on request.
