# Supplementary material for: ROS-lowering doses of vitamins C and A accelerate malignant melanoma metastasis
Source: Redox Biol. 2023 Feb 2;60:102619. doi: 10.1016/j.redox.2023.102619 (PMC9945759; doi:10.1016/j.redox.2023.102619)

SUPPLEMENTARY FIGURE LEGENDS

Supplementary Figure 1. Examples of IncuCyte images for defining migration enhancers and migration suppressors. Related to Figure 1.

IncuCyte images were taken before (first image) and following incubation with a redox active compound (treatment) for 24 h (last image). Control cells were incubated with the same volume of DMSO. The migration enhancer closed the scratch wound more efficiently than control, the migration suppressor less efficiently.

Supplementary Figure 2. Screening 104 redox-active compounds for their ability to influence melanoma cell migration. Related to Figure 1.

SK-MEL-30 cells were incubated with 104 redox active compounds as outlined in Figure 1A to test the impact on cell migration using a wound-healing assay. The cells were incubated with two concentrations (50 µM, blue horizontal bars; and 5 µM, red bars) of the 104 compounds. The horizontal bars from zero to the right represent increased migration compared with DMSO control; bars from zero to left represent decreased migration. Stars at left indicate the 18 compounds that increased migration at both doses.

Supplementary Figure 3. Common antioxidants do not influence primary tumor growth in mice with BRAF^{V600E}-induced malignant melanoma. Related to Figure 2A.

Primary tumor size in *Braf*$^{CA/+}$*Pten*$^{fl/fl}$Tyr-*Cre*$^{+/0}$ mice painted with tamoxifen at 2 days of age and randomized at weaning to receive: control food and drinking water; drinking water supplemented with vitamin C; or food supplemented with canthaxanthin, β-carotene, or retinyl palmitate. Each compound was administered in two different doses as indicated.

Supplementary Figure 4. RNAseq analyses and knocking out *Bach1* in mouse B16-F1 melanoma cells and the impact of these on survival following injection into syngeneic mice. Related to Figure 2, B–F.

(**A**) The top enriched pathways from analyses of RNAseq data of human malignant melanoma cells from experiments shown in Figure 2, B and C. (**B**) log2Fold-changes in the expression of four GSH-related genes. (**C**) Schematic of *Bach1*'s 5 exons (rectangles, coding sequence in grey) and introns/non-coding sequences (lines). Arrowhead indicates the sgRNA-Cas9 cut site in exon 2; arrows indicate primers used for PCR identification and sequencing of targeted clones. (**D**) Chromatogram of the $Bach1^{+/+}$ (WT) and $Bach1^{-/-}$ (KO) clones. Arrowhead indicates sgRNA-Cas9 cut site. (**E**) Western blot of whole protein extracts with antibodies recognizing BACH1. ACTIN was the loading control. (**F**) Survival of mice injected with $Bach1^{+/+}$ and $Bach1^{-/-}$ B16F1 melanoma cells from the experiment in Figure 2, E and F.

Supplementary Figure 1

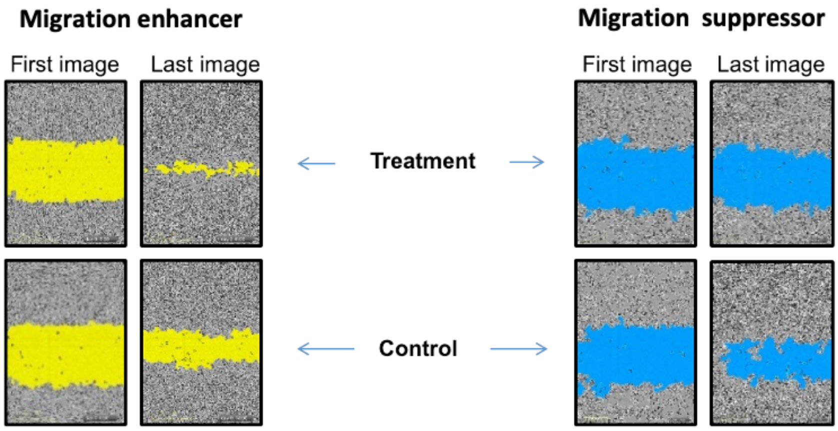

Supplementary Figure 2

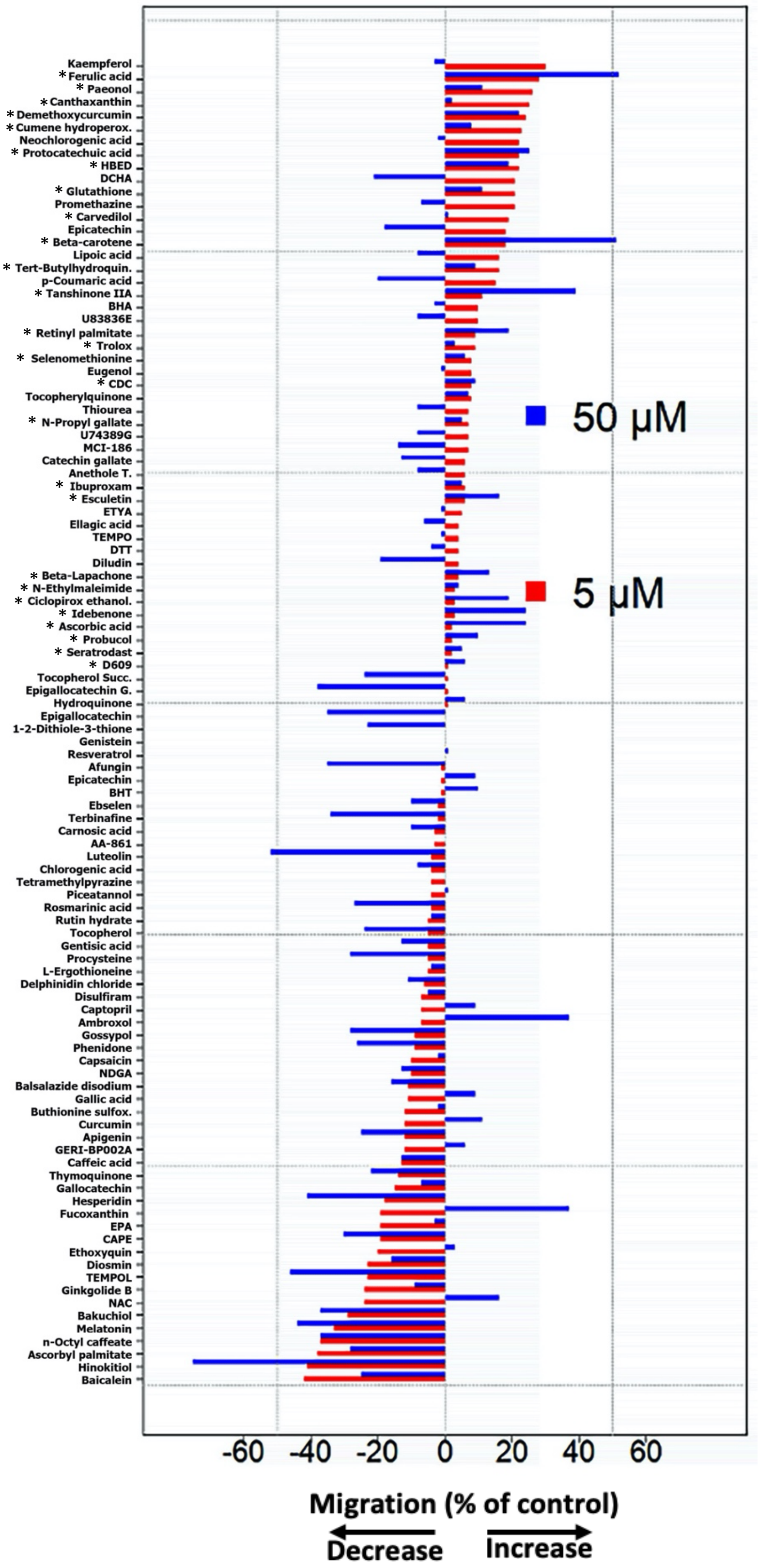

Supplementary Figure 3

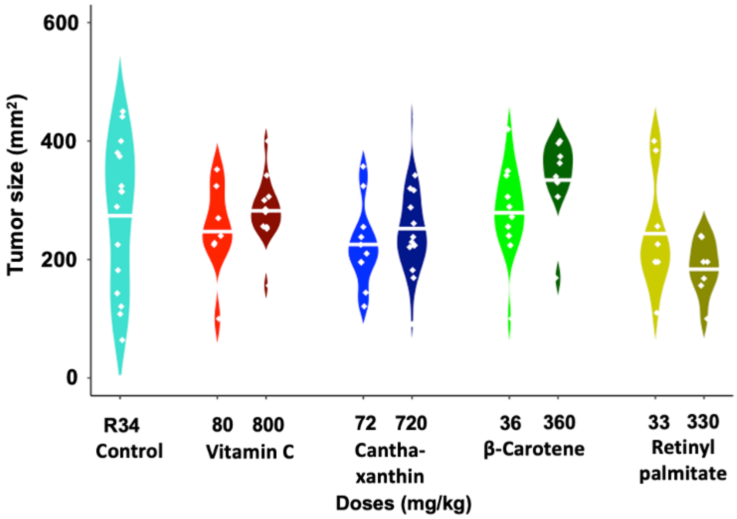

Supplementary Figure 4

A

Pathways	p value
Nuclear receptors meta-pathway	5.5×10^{-9}
Photodynamic therapy induced NRF2 survival signaling	1.7×10^{-6}
NRF2 pathway	1.6×10^{-5}
Nuclear receptors in lipid metabolism and toxicity	1.1×10^{-3}
Retinoblastoma gene in cancer	3.7×10^{-3}

B

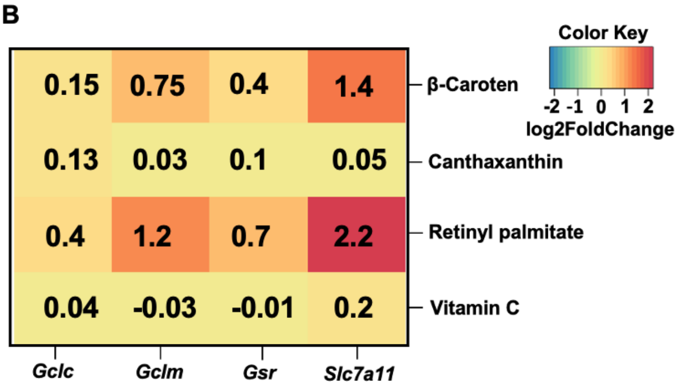

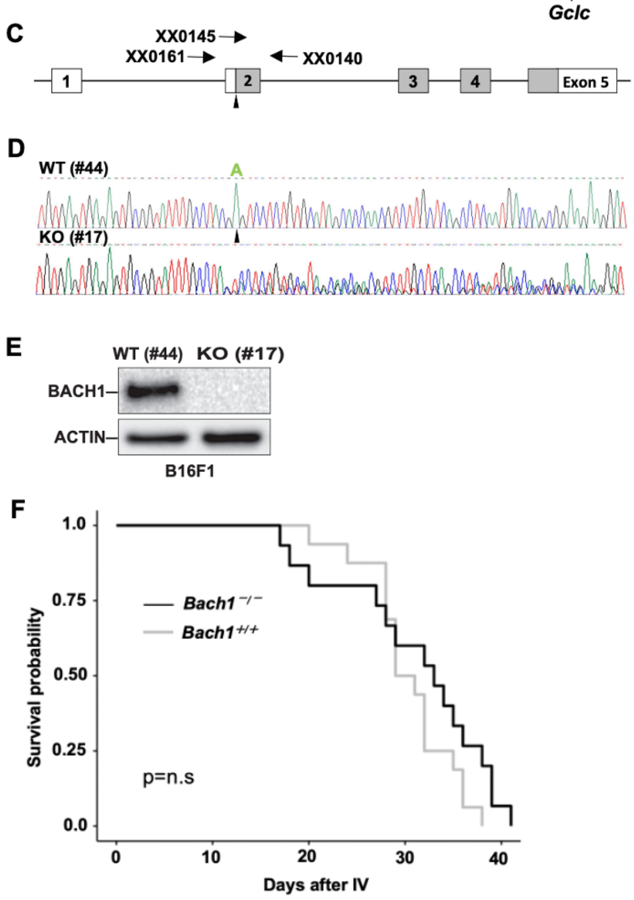

Supplement: Multimedia component 1 [file mmc1.pdf]
